# Leishmaniasis: Middle East and North Africa Research and Development Priorities

**DOI:** 10.1371/journal.pntd.0001219

**Published:** 2011-07-26

**Authors:** Mary Ann McDowell, Sima Rafati, Marcelo Ramalho-Ortigao, Afif Ben Salah

**Affiliations:** 1 Eck Institute for Global Health, Department of Biological Sciences, University of Notre Dame, Notre Dame, Indiana, United States of America; 2 Molecular Immunology and Vaccine Research Laboratory, Pasteur Institute of Iran, Tehran, Iran; 3 Department of Entomology, Kansas State University, Manhattan, Kansas, United States of America; 4 Pasteur Institute of Tunis, Laboratory of Medical Epidemiology, Tunis, Tunisia; Yale School of Public Health, United States of America

## Introduction

*“Science knows no country, because knowledge belongs to humanity, and is the torch which illuminates the world.”—*Louis Pasteur

**Figure 1:**
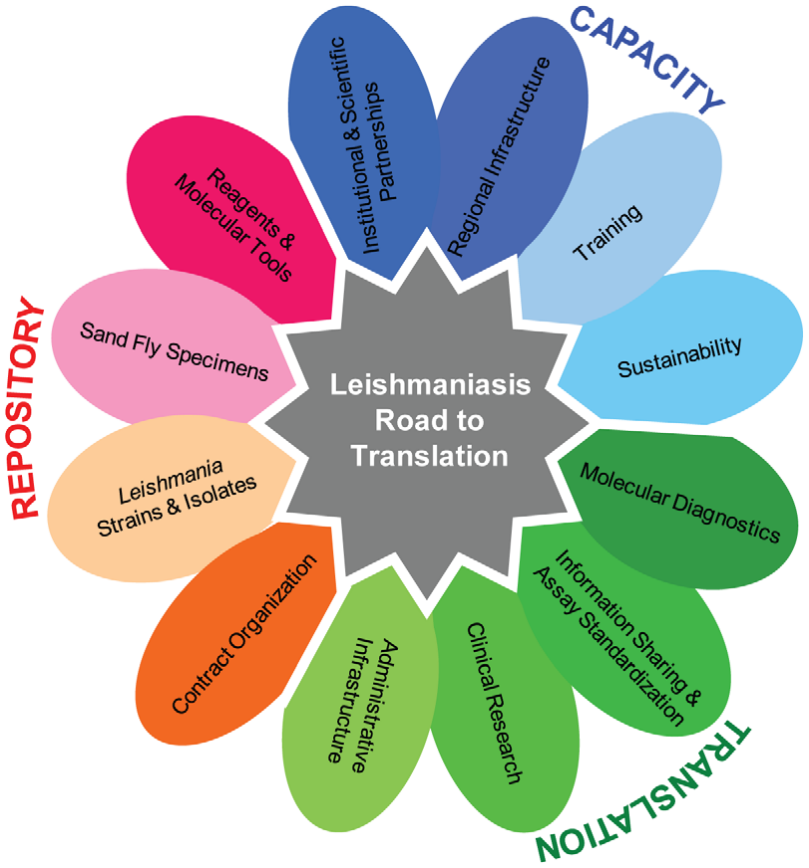
Policy recommendations for the translation of laboratory discoveries into field applications for the control of leishmanisis.

Leishmaniasis remains one of the world's most devastating neglected tropical diseases, causing substantial mortality and contributing to nearly 2 million disability-adjusted life years. The true global burden of leishmaniasis, however, is unknown. The Middle East and North Africa (MENA) region is endemic for many forms of leishmanisis and has hosted many recent epidemic outbreaks. A research and policy conference, LEISHMANIA: Collaborative Research Opportunities in North Africa and the Middle East, was held in June 2009 in Tunisia to promote international collaboration between the United States (US) and the countries most affected by Old World leishmaniasis (see Table 1 for a list of participating countries). Supported by the US National Institute of Allergy and Infectious Diseases, National Institutes of Health (NIH), and hosted locally by the Institute Pasteur de Tunis, approximately 100 scientists and administrators from the US and MENA countries met to share critical information and to identify the major obstacles for translating scientific breakthroughs into innovative strategies for reducing the burden of leishmaniasis. The participants identified three crucial areas as requiring reinforcement and growth: translation of laboratory discoveries into field-applications, increased research capacity in endemic countries, and the creation of a leishmaniasis reagent repository (Figure 1). Our hope is that these recommendations will be adopted by research, funding, and policy institutions alike to have a greater impact at controlling leishmaniasis throughout the world.

**Table 1:** Countries Represented.

Country	Scientists
Afghanistan	1
Algeria	1
Egypt	4
Iran	3
Jordan	1
Lybia	1
Mali	1
Morocco	2
Palestinian Authority	2
Sudan	8
Syria	1
Tunisia	30
Turkey	1
United States of America	26

## Leishmaniasis in the Middle East and North Africa

**Figure 2:**
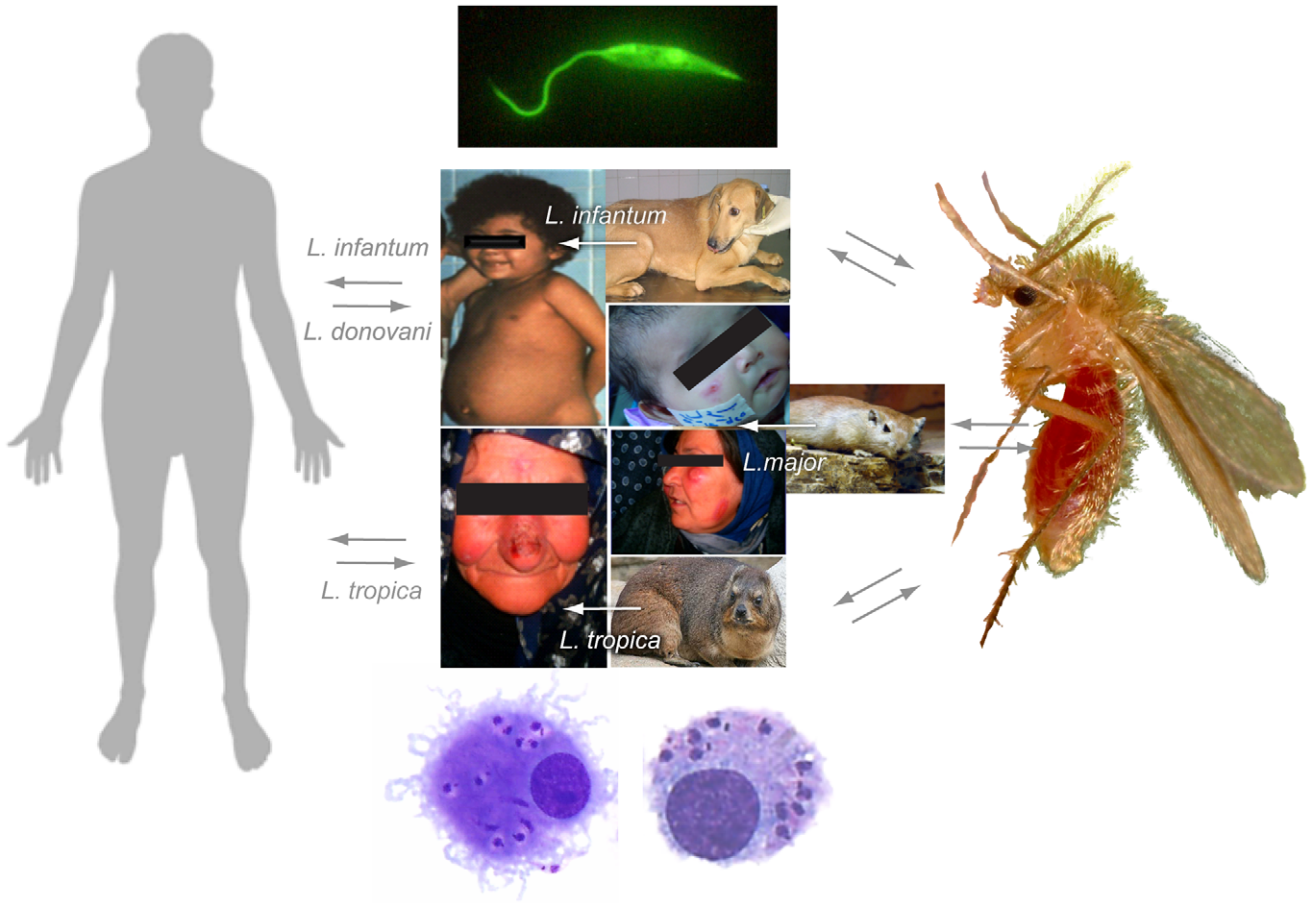
MENA leishmaniasis. Although at least 20 *Leishmania spp.* infect humans worldwide, the primary epidemiologically relevant species in the MENA region are *L. major*, *L. tropica*, *L. infantum*, and *L. donovani*, transmitted by approximately 25 different *Phlebotomus spp*. Etiological agents of visceral leishmaniasis (VL) in MENA include *L. donovani*, *L. infantum*, and occasionally *L. tropica*. Cutaneous leishmaniasis (CL) caused by *L. major*, *L. tropica*, and *L. infantum* differ slightly in lesion presentation depending on the species. As with vector species, a variety of animal hosts have been implicated as reservoirs in the transmission of zoonotic leishmaniasis, including rodents, hyraxes, and canids. For CL caused by *L. major*, the primary cycle is zoonotic between *P. papatasi* (shown) and *Psammoys* (shown) and *Meriones* rodents. Although hyraxes have been implicated as a reservoir host for *L. tropica*, transmission is thought to be primarily anthroponotic as is the VL agent, *L. donovani*. Mediterranean VL caused by *L. infantum* is typically zoonotic where candids are the primary reservoir and man is an accidental host; however, anthroponitic cycles also have been characterized. Regardless of species or clinical manifestation, all *Leishmania* species infecting humans are transmitted by the bite of an infected sand fly. During a blood meal, metacyclic promastigotes are released by the sand fly and enter the skin of the vertebrate host. *Leishmania* parasites infect cells of the myeloid lineage, including neutrophils, followed by macrophages and dendritic cells (shown). These parasites reside within a phagolysosome where they differentiate into a dividing, aflagellated amasitogotes. Sand flies take up parasites when feeding on an infected host. Infected host cells are lysed and amastigotes differentiate into flagellated procyclic promastigotes that attach to the midgut of the sand fly vector. Subsequent development and migration towards the anterior end of the sand fly completes the cycle. Photo Credits: *P. papatasi* courtesy of Tim Gathany, Center for Disease Control Photo Services; *Psammomys obesus* from http://commons.wikimedia.org/wiki/File:Psammomys_obesus_01.jpg; rock hyrax from http://www.marietta.edu/~biol/biomes/biome_main.htm.

Leishmaniasis is a complex, multi-spectrum, sand fly–transmitted disease (Figure 2), with symptoms ranging from self-limiting cutaneous lesions to fatal visceral disease. While the species of *Leishmania* initiating the infection primarily dictates clinical presentation, a single *Leishmania* species can elicit a range of pathologies [1], [2], [3]. The specific factors that influence clinical outcome of these infections remains to be completely elucidated, but likely is influenced by host genetics or immune responses [4], potentially different sand fly vector species or populations [5], [6], [7], and/or the presence of *Leishmania spp.* hybrids [8], [9].

Although the World Health Organization (WHO) has designated leishmaniasis as one of the 15 most neglected tropical diseases, the global morbidity due to leishmaniasis remains underestimated due to misdiagnosis and inadequate reporting guidelines. In the MENA region alone, nearly 100,000 cases of leishmaniasis in 2008 were reported to the WHO; however, some nations only report for one of the various clinical pathologies, and other countries fail to report all together [10]. Epidemics of leishmaniasis often are associated with war, human migration, and anthroponotic environmental change associated with agricultural practices or urbanization. The key factors driving the risk, severity, and temporal-spatial trends of disease, however, are presently unknown, and predictive tools for epidemic peaks and geographic spread of leishmanial disease are lacking [11].

## Translation of Laboratory Discoveries into Field Applications

*“Science and the applications of science bound together as the fruit of the tree which bears it.” —*Louis Pasteur

Science is in the midst of a technological explosion, and the next wave of scientific progress will involve translating these advancements into practical implementation policies in disease-endemic regions. Many novel techniques are furthering our understanding of various aspects of *Leishmania* transmission, disease, and sand fly biology, tools that hold promise for bridging the bench to the bedside. Unfortunately, this revolution has not translated into meaningful control measures. Advancement in three major priority areas, molecular diagnostics, clinical research, and knowledge sharing and standardization, is necessary to push the leishmaniasis field towards translational results (Table 2).

**Table 2:** Leishmaniasis: Road to Translation.

Translation of Laboratory Discoveries to Field Application		
Molecular Diagnostics	Clinical Research	Information Sharing
*Leishmania* Species	Administrative Infrastructure	Assay Standardization
Sand Fly Species	Best Practices Guidelines	Digital Libraries
Genomics Research	Streamlined HRP/MTA/NDA	Data Sharing

Capacity Building		
Partnerships	Training	Infrastructure
Institutional	PhD Sandwich Programs	Laboratory Facilities
Scientific Collaborations	Regional Workshops	Administrative
Seed Funding/Travel Grants	Web-based Courses	Regional Meetings

Repository		
*Leishmania*	Sand Flies	Reagents
Clinical Isolates	Type Specimens	Hybridomas
Parasite Strains	Tissues	Genomic Resources
		Molecular Tools

### Molecular Diagnostics

Specific parasite identification methods are essential if the complexities of leishmanial disease manifestation are to be elucidated; to effectively impact epidemiological studies as well as clinical treatment, these diagnostic tools must be reliable, inexpensive, easy to use, and provide quick results. Comparative genome analyses of completed *Leishmania* genomes will greatly enhance development of novel molecular approaches to identify different *Leishmania* species and strains, and as next generation sequencing methods become more affordable, genome sequence information will be more easily obtainable for clinical isolates. Bolstering genomics research in general and increasing genomics capacity in regional environments is crucial if we hope to unravel the complex dynamics of leishmaniasis.

Molecular tool development also is essential to produce a complete epidemiological picture of vector distributions and how these species correlate with the current state of leishmaniasis in the MENA region. New insights concerning sand fly biology will likely be provided with the completion of the ongoing genome projects of two sand fly species, *Phlebotomus papatasi* and *Lutzomyia longipalpis*.

### Clinical Research

Development of a vaccine to prevent leishmaniasis has been motivated by the observation that a cured infection protects against re-infection. While laboratory studies and anecdotal evidence indicate that “natural infection” protects from severe disease, true validation in the field is necessary, as cases of re-infection have been reported [12], [13]. Model systems certainly can provide critical information, but they never truly mimic the disease dynamics in nature. Although delineating the mechanisms that lead to parasite establishment and tissue-specific immunity in humans remains an ethical challenge, clinical research in leishmaniasis-endemic regions needs to be expanded. In addition to the evaluation of vaccine trials and anti-leishmanial drugs, implementation of large, longitudinal, cohort studies will improve the understanding of that natural history of *Leishmania* infection and its transmission dynamics, as well as the parameters that lead to the wide disease spectrum.

Regulatory bottlenecks due to inadequate administrative frameworks often hinder clinical research activity in disease-endemic countries. The international research community could bridge this infrastructure gap by facilitating clear and coordinated international human subjects' research protection (HRP) policy conformity through streamlined procedures for establishing and registering Institutional Review Boards and Federalwide Assurance compliance in all nations. Furthermore, research institutions and private industry should be encouraged to establish policies for the ease of executing material transfer agreements (MTA) and non-disclosure arrangements (NDA) in low- and middle-income countries.

### Knowledge Sharing and Standardization

Rapid advances are being made in the leishmaniasis field, some of which are opening new avenues for investigation and hold promise for translational application. For these breakthroughs to be effective, however, technology needs to be efficiently transferred to regional scientists. Not only is knowledge sharing essential between scientists of resource-rich and low- and middle-income nations, there also is a dire necessity for assay standardization between the entire leishmaniasis research community. Clear guidelines and best practices should be created for assessing vaccine efficacy and validating chemotherapies.

Also essential is the generation of guidelines for the development and implementation of field-based studies and clinical trials. Standardization of experimental design, reporting statistics, and clinical protocols will be required across nations to adequately derive conclusions from multiple investigations. Furthermore, development of protocols to assess pertinence, effectiveness, and robustness of surveillance programs are desperately needed.

Successful implementation of these clinical studies will only advance the leishmaniasis field if critical information is shared among researchers, policy makers, and practitioners. Timely information dissemination will be required as the leishmaniasis field continues to expand; such exchange could appear in public digital libraries, through development of secure Web sites for clinical data, or by implementation of smaller regional meetings that foster interaction between scientists, clinicians, and government personnel.

## Capacity Building

Increased investments in research, availability of laboratory facilities, and close collaboration among scientists would greatly advance the leishmaniasis field. Strengthening the connections between education, research, and society are essential factors and require certain building blocks to assure its proper structure. However, in resource-poor locations it is difficult to establish and sustain such cooperative enterprises. This arduous task requires global capacity building in science and technology, and active participation of the international scientific community could have tremendous effects.

The persistent loss of national scientific talent is an important issue that low- and middle-income countries are facing and is a major obstacle to the proper establishment of research activities in endemic countries. In order to be successful, scientific institutions need access to infrastructure, technical support, and trained scientists. By creating a thriving scientific atmosphere, researchers will be retained and motivated. In order to increase scientific capacity in the MENA region, focused efforts in the following areas are necessary:

1. Long-term partnerships between scientists and institutions, including both academic and public health organizations, must be fostered and supported by funding agencies, governments, and private and public institutions. Proactive actions should to be taken to develop mutually advantageous collaborative projects through directed seed funding.
2. Development of institutional partnerships with lasting student and faculty exchange programs. Sandwich training of PhD students is particularly useful at bringing and retaining regional scientists.
3. The organization of training workshops and courses can serve as a mechanism of active collaboration among different institutes. These training modules should be located in leishmaniasis-endemic countries for greater accessibility to scientists from these areas.
4. Creation of Web sites where subject-specific teaching materials and assay protocols are easily accessed.
5. Availability of visiting lectureships and travel grants for participation in international scientific meetings to scientists, post-graduate students, and technicians from disease-endemic countries should be increased.
6. Establishment and support of local and international scientific associations that meet on a regular basis would increase information sharing among endemic scientists and policy makers.

## Creation of a *Leishmania/*Sand Fly Reagent Repository

Another priority area that will increase the research capacity in leishmaniasis-endemic countries is the creation of a centralized leishmaniasis reagent repository. An enterprise such as the Malaria Research and Reference Reagent Resource Center (MR4) housed at the American Type Culture Collection (ATCC) would greatly enhance the availability of leishmanisis reagents to resource-poor nations. Genomic databases, like TriTypDB (http://tritrypdb.org/) for kinetoplastid organisms, EuPathDB (http://eupathdb.org/) for eukaryotic pathogens, and VectorBase (http://www.vectorbase.org/) for arthropod vectors of disease, that manage genmic data and support centralized bioinformatics tools have already provided essential resources to researchers from disease-endemic countries. The next critical step, however, is for the genomic reagents connected to genome projects to be readily accessible to the international research community. In addition to genomic tools, a depot stocking *Leishmania* strains and mutants, clinical isolates, tissue samples, and other leishmaniasis research reagents would improve the access of such materials to scientists worldwide. One obstacle to effectively controlling leishmaniasis is an inadequate picture of the sand fly strains that can transmit *Leishmania* parasites. As molecular systematics is a rapidly evolving field, type specimens for future analysis also could be stored and distributed by a leishmaniasis reagent center.

In addition to increased access of reagents to international scientists, an added benefit of a leishmaniasis repository would be the availability of *Leishmania* strains and other reagents for verification by multiple laboratories and additional experimental platforms. Moreover, easy access of materials might encourage scientists from other disciplines to apply their expertise to the leishmaniasis field, bringing fresh ideas and approaches to controlling these destructive diseases.

There are currently limited *Leishmania* repositories in the United Kingdom, France, and Brazil, as well as sand fly collections housed by universities and museums in different nations. However, no centralized location exists that curates, stores, and distributes these resources to the entire international research community. As leishmaniasis research continues to expand, the financial cost to these smaller storage facilities may become too much of a burden. International collaborative leishmaniasis research would be greatly strengthened if a sustained leishmaniasis repository was created and ease the cost and difficulty of the distribution of scientific samples across international borders. Clearly, such an enterprise would require oversight and funding, and strict guidelines for sample submission and quality control must be implemented. Like MR4, which is supported by NIH and serves the malaria community, such a facility could be funded by a collection of public and private agencies and would be best developed through a competitive bid process.

## Summary

The US-MENA Leishmaniasis conference provided an unprecedented opportunity for scientists to interact and focus on leishmaniasis research issues specific to the MENA region and resulted in several successful grant proposals funded through the Civilian Research and Development Foundation. The collaborations that arose from this conference will certainly influence leishmaniasis control in the MENA region specifically; moreover, the discussions and recommendations also have implications for the leishmaniasis field as a whole.
